# Identifying Regulators of MHC-II Presentation in Lung Epithelial (BEAS-2B) Cells via a Forward Genetic Screen

**DOI:** 10.51894/001c.143841

**Published:** 2025-09-30

**Authors:** Mehdi A. Abed, Andrew J. Olive

**Affiliations:** 1 College of Osteopathic Medicine Michigan State University; 2 College of Osteopathic Medicine Michigan State University

## 07

### Introduction

Antigen presentation via Major Histocompatibility Complex Class II (MHC-II) is critical for CD4+ T-cell activation and immune homeostasis. While MHC-II regulation in professional antigen-presenting cells (APCs) is well characterized, its regulation in non-APC cells like epithelial cells remains unclear. Lung epithelial cells express MHC-II in response to Interferon gamma (IFN-γ), produced by activated Th1 cells during infections or perturbations. MHC-II expression modulates T-cell activation, and its dysregulation contributes to respiratory diseases such as infections, asthma, and chronic pulmonary disease.

### Objectives

Understanding the regulation of MHC-II on lung epithelial cells is critically important to define key regulators of pulmonary inflammation and help identify new potential therapeutic targets to improve lung homeostasis during health and disease. We hypothesize that lung epithelial cells use distinct regulatory mechanisms than those used in professional APCs to control the expression of MHC-II.

### Methods

To test this hypothesis, we conducted a forward genetic screen to dissect MHC-II regulation in human bronchial epithelial cells (BEAS-2B cells). We generated a genome-wide CRISPR-Cas9 knockout library and then stimulated the library with IFN-γ to induce MHC-II expression. We isolated knockout cell populations with high or low surface expression levels of MHC-II. We then sequenced the sgRNAs in these sorted cells and using MaGECK analysis identified positive and negative regulators of MHC-II surface expression.

### Results

Results revealed significant enrichment of known genes involved in MHC-II expression and IFN-γ response among the MHC-II low population, validating our approach. Notably, we identified novel candidates, including components of the COP9 signalosome complex, a regulator of Cullin-RING ubiquitin ligases suggesting a previously unrecognized role for this complex in immune regulation.

### Conclusion

We are validating additional candidate genes and investigating their roles in transcriptional activation and expression of MHC-II on the cell surface. Future studies include how these candidates influence T-cell interactions and effector functions. These findings will uncover unique regulatory pathways in lung epithelial cells critical for antigen presentation and immune modulation. By identifying novel regulators of MHC-II expression in lung epithelial cells, this work provides a foundation for potential therapeutic targets to modulate pulmonary inflammation and restore lung homeostasis during health and diseases.

